# Mice lacking *Casp1*, *Ifngr* and *Nos2* genes exhibit altered depressive- and anxiety-like behaviour, and gut microbiome composition

**DOI:** 10.1038/s41598-018-38055-8

**Published:** 2019-04-23

**Authors:** Antonio Inserra, Jocelyn M. Choo, Martin D. Lewis, Geraint B. Rogers, Ma-Li Wong, Julio Licinio

**Affiliations:** 1 Mind and Brain Theme, South Australian Health and Medical Research Institute, Adelaide, SA Australia; 2 Discipline of Psychiatry, College of Medicine and Public Health, Flinders University, Bedford Park, SA Australia; 3 Centre for Neuroscience, Flinders University, Bedford Park, Australia; 4 Infection and Immunity Theme, South Australia Health and Medical Research Institute, North Terrace, Adelaide, SA Australia; 5 SAHMRI Microbiome Research Laboratory, Flinders University College of Medicine and Public Health, Bedford Park, SA Australia; 6 State University of New York Upstate Medical University, Syracuse, NY USA

**Keywords:** Target identification, Stress and resilience, Depression, Molecular medicine

## Abstract

Converging evidence supports the involvement of pro-inflammatory pathways and the gut microbiome in major depressive disorder (MDD). Pre-clinical and clinical studies suggest that decreasing pro-inflammatory signaling may provide clinical benefit in MDD. In this study, we used the chronic unpredictable stress (CUS) paradigm to assess whether mice lacking the pro-inflammatory caspase 1, interferon gamma-receptor, and nitric oxide synthase (*Casp1, Ifngr, Nos2*)^−/−^ present altered depressive- and anxiety-like behaviour at baseline and in response to CUS. In comparison to wild-type (wt) mice, (*Casp1, Ifngr, Nos2*)^−/−^ mice displayed decreased depressive- and anxiety-like behaviour, and increased hedonic-like behaviour and locomotor activity at baseline, and resistance to developing anhedonic-like behaviour and a heightened emotional state following stress. Plasma levels of ACTH and CORT did not differ between the triple knockout and wt mice following stress. The faecal microbiome of (*Casp1, Ifngr, Nos2*)^−/−^ mice differed from that of wt mice at baseline and displayed reduced changes in response to chronic stress. Our results demonstrate that simultaneous deficit in multiple pro-inflammatory pathways has antidepressant-like effects at baseline, and confers resilience to stress-induced anhedonic-like behaviour. Moreover, accompanying changes in the gut microbiome composition suggest that CASP1, IFNGR and NOS2 play a role in maintaining microbiome homeostasis.

## Introduction

Increasing evidence implicates neuroinflammatory pathways in the development, treatment response, and remission of MDD^1,2^. Dysregulation of three major inflammatory systems is evident in those patients: (a) increased oxidative stress by means of nitric oxide (NO) overproduction, driven by NOS2 (NO synthase 2 or inducible NOS)^3,4^, (b) low-grade chronic pro-inflammatory status driven by CASP1 overproduction^5,6^, and (c) INFG over production driven by Th1 lymphocytes^7–9^.

Each of these pathways has been investigated in isolation as an antidepressant-like strategy for MDD^10–12^. However, the complex nature of MDD pathophysiology and the potential interplay between pathways suggests that multi-targeted pharmacotherapeutic approaches might provide more significant benefit than isolated pathway approaches. To investigate the effects of multiple pro-inflammatory mediator deficiencies at baseline and post-stress behaviour, we generated a triple knockout mouse model lacking *Casp1*, *Ifngr* and *Nos2* (*Casp1, Ifngr, Nos2*)^−/−^, and assessed them in a pre-clinical paradigm of stress-induced depressive-like behaviour.

Immune regulation contributes to the maintenance of gut microbiome homeostasis^13^. Therefore, changes in inflammatory states are likely to give rise to shifts in gut microbiota composition, potentially contributing to MDD development through a variety of pathways^10,14,15^. Also, emerging studies implicate the gut microbiota in the regulation of stress responses^16,17^, while depressed patients display abnormal faecal microbiota composition associated with heightened inflammatory signalling^5,7,18–21^.

We therefore further assessed whether: 1) the (*Casp1, Ifngr, Nos2*)^−/−^ model is associated with an altered basal gut microbiota composition compared with wt, and 2) (*Casp1, Ifngr, Nos2*)^−/−^ mice are protected from stress-induced shifts in gut microbiome composition, due to deficits on mediating inflammatory pathways.

## Results

### *Casp1*, *Ifngr* and *Nos2* deficiency decreases depressive-like and anxiety-like behaviour

**Figure 1:**
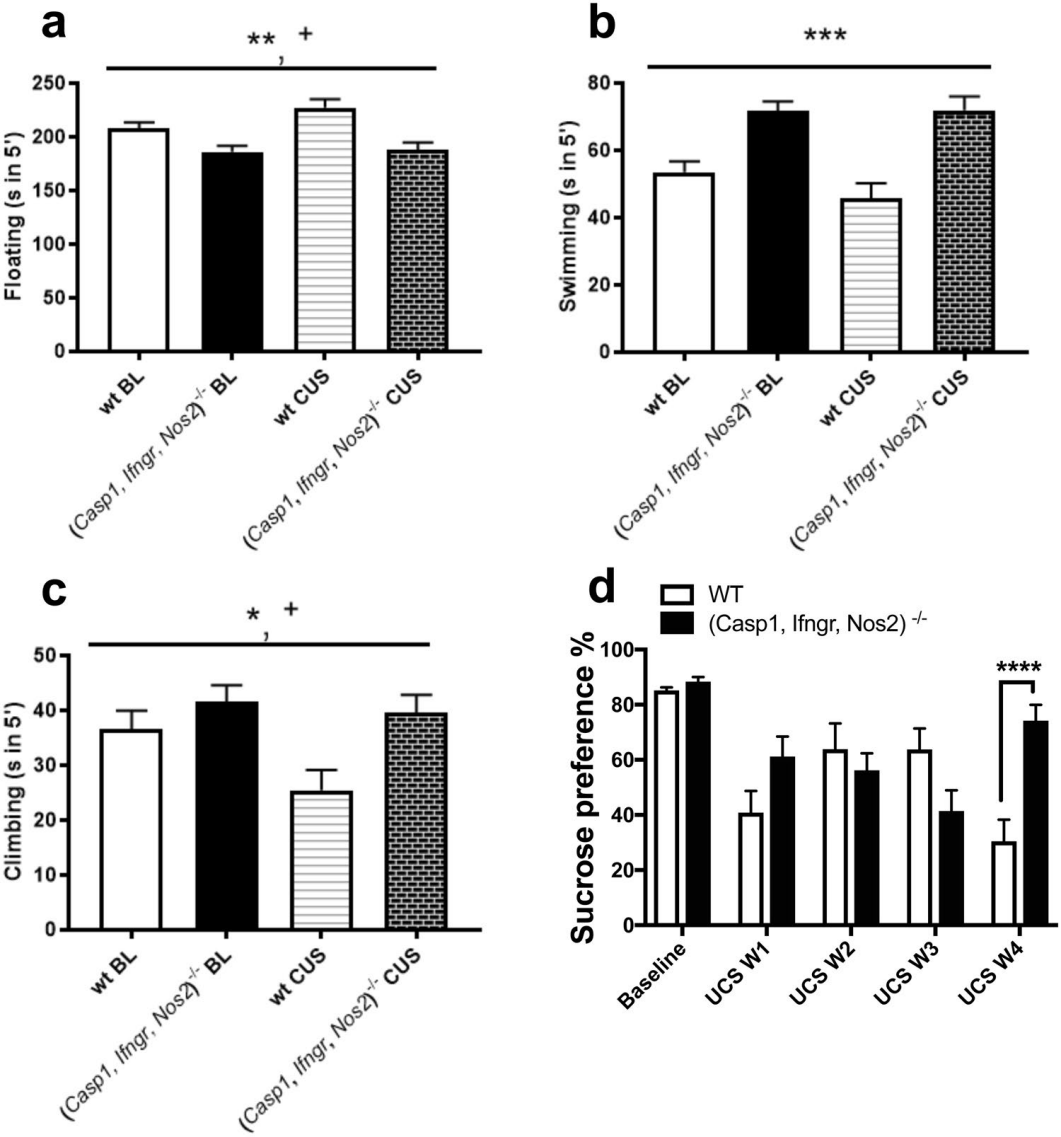
*Casp1*, *Ifngr* and *Nos2* deficiency decreases depressive-like behaviour. (**a**) (*Casp1, Ifngr, Nos2*)^−/−^ mice displayed decreased floating time in the forced swim test in comparison to wild-type (wt) mice while displaying (**b**) increased swimming and (**c**) climbing behaviours. Moreover, (**d**) (*Casp1, Ifngr, Nos2*)^−/−^ mice displayed an increased preference for a 1% sucrose solution. Data are presented as means ± s.e.m. Genotype effect *P < 0.05; **P < 0.01; ***P < 0.001; stress effect ^+^*P* < 0.05; ^++^*P* < 0.01; ^+++^*P* < 0.001; genotype*stress effect ^#^*P* < 0.05; ^##^*P* < 0.01; ^###^*P* < 0.001. wt = wild-type; BL = baseline; CUS = after unpredictable chronic stress paradigm; wt n = 16; (*Casp1, Ifngr, Nos2*)^−/−^ n = 20.

**Figure 2:**
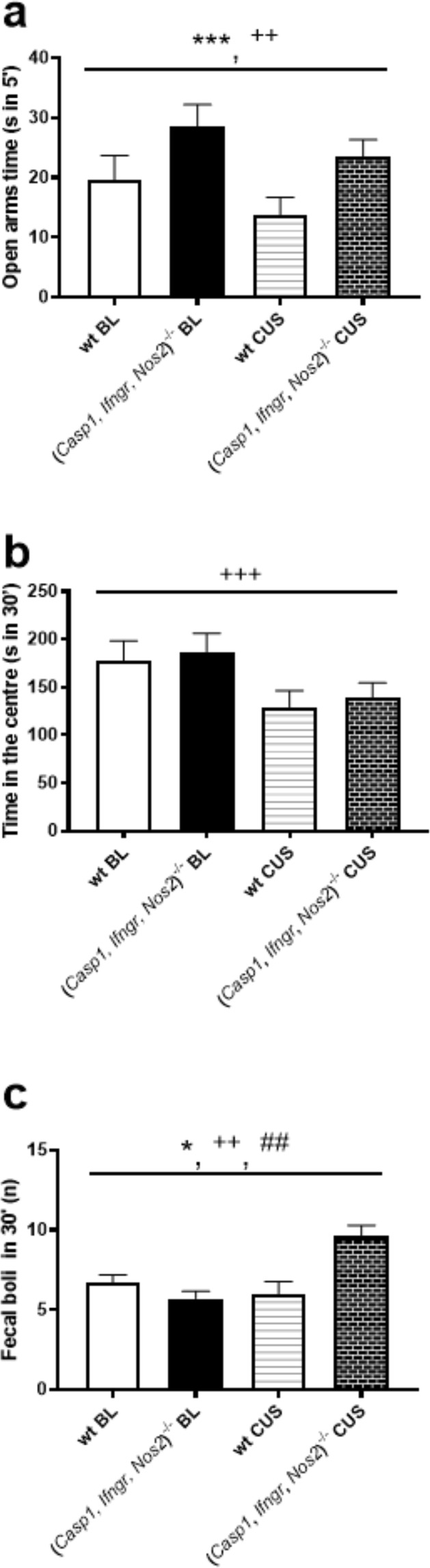
*Casp1*, *Ifngr* and *Nos2* deficiency decreases anxiety-like behaviour. (**a**) (*Casp1, Ifngr, Nos2*)^−/−^ mice displayed increased time spent in the open arms of the elevated plus maze but (**b**) similar time in the centre area of the open field test. The (*Casp1, Ifngr, Nos2*)^−/−^ genotype had a main effect on (**c**) the number of defecations during the open field test, which was driven by the increased number of faecal boli in (*Casp1, Ifngr, Nos2*)^−/−^ mice following CUS, while this parameter remained unchanged in wt mice. Data are presented as means ± s.e.m. Genotype effect *P < 0.05; **P < 0.01; ***P < 0.001; stress effect ^+^*P* < 0.05; ^++^*P* < 0.01; ^+++^*P* < 0.001; genotype*stress effect ^#^*P* < 0.05; ^##^*P* < 0.01; ^###^*P* < 0.001; wt = wild-type; BL = baseline; CUS = chronic unpredictable stress paradigm; wt n = 16; (*Casp1, Ifngr, Nos2*)^−/−^ n = 20.

Total floating time in the forced swim test was lower in (*Casp1, Ifngr, Nos2*)^−/−^ mice compared to wt mice (F_1,34_ = 14.618, *P* = 0.001) (Fig. 1a and Supplementary Table 1). Swimming and climbing behaviours were increased in (*Casp1, Ifngr, Nos2*)^−/−^ mice compared to wt mice (respectively F_1,34_ = 25.256, *P* = 0.001, Fig. 1b and F_1,34_ = 5.929, *P* = 0.020, Fig. 1c). Similarly, preference for a 1% sucrose solution in the sucrose preference test was increased in the (*Casp1, Ifngr, Nos2*)^−/−^ genotype compared to wt mice (F_1,34_ = 23.331, *P* < 0.001) (Fig. 1d). Moreover, (*Casp1, Ifngr, Nos2*)^−/−^ mice spent more time in the open arms of the elevated plus maze compared to wt mice (F_1,34_ = 15.480, *P* < 0.001) (Fig. 2a) and displayed an increased open/closed arms entries ratio (F_1,34_ = 17.302, *P* < 0.001); in contrast, no differences were observed in the total time spent in the centre of the open field arena between (*Casp1, Ifngr, Nos2*)^−/−^ and wt mice (F_1,34_ = 0.200, *P* = 0.658) (Fig. 2b) and in the ratio centre/total distance in the open field test (F_1,34_ = 3.330, P = 0.077). In the open field test, the (*Casp1, Ifngr, Nos2*)^−/−^ genotype was associated with a higher number of faecal boli (F_1,34_ = 4.128, *P* = 0.050) (Fig. 2c).

### *Casp1*, *Ifngr* and *Nos2* deficiency affects the response to chronic unpredictable stress (CUS)

Following 28 days of CUS, the preference for a 1% sucrose solution in the sucrose preference test varied as a function of genotype (F_1,34_ = 17.485, *P* < 0.001) (Fig. 1d). Wt mice showed a ~74% decrease in sucrose preference compared to baseline (F_1,34_ = 57.25 *P* < 0.001), while (*Casp1, Ifngr, Nos2*)^−/−^ mice showed a decrease of ~16% (F_1,34_ = 4.78, *P* = 0.036), reaching an after-stress sucrose preference of 74.2%.

**Figure 3:**
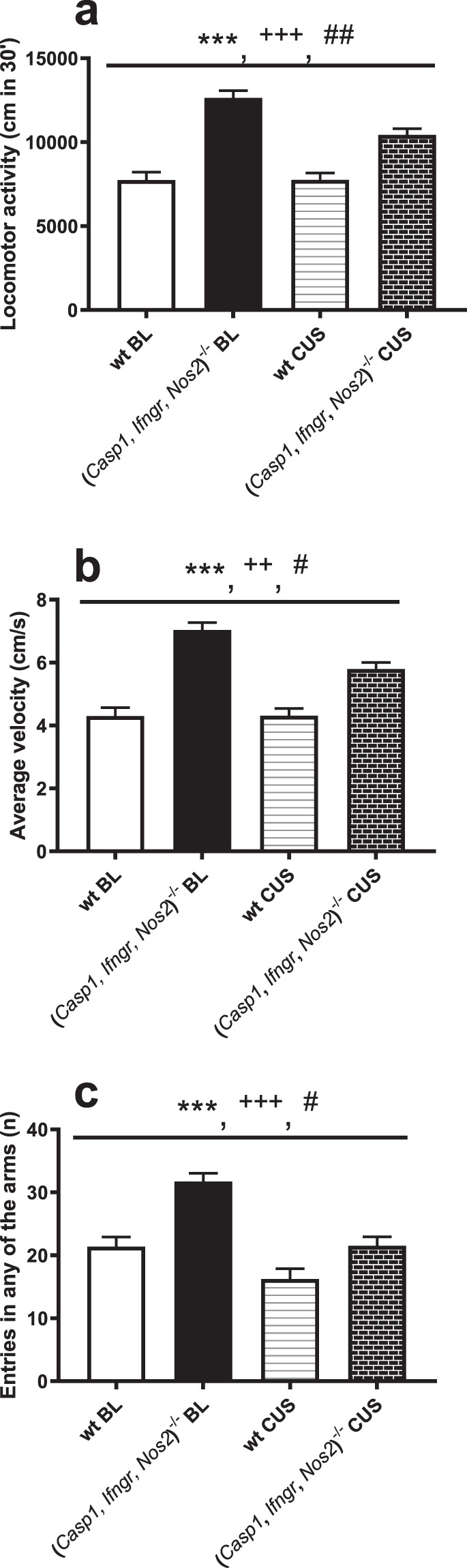
*Casp1*, *Ifngr* and *Nos2* deficiency increases locomotor activity. (**a**) (*Casp1, Ifngr, Nos2*)^−/−^ mice displayed increased locomotor activity and (**b**) average moving velocity in the open field test. Moreover, (**c**) (*Casp1, Ifngr, Nos2*)^−/−^ genotype had a main effect on the total number of entries in any of the arms of the elevated plus maze, irrespective of them being open or closed. All those parameters showed a stress-genotype interaction, with a common trend of being decreased as a result of *Casp1, Ifngr* and *Nos2* deficiency following chronic unpredictable stress (CUS). Data are presented as means ± s.e.m. Genotype effect *P < 0.05; **P < 0.01; ***P < 0.001; stress effect ^+^*P* < 0.05; ^++^*P* < 0.01; ^+++^*P* < 0.001; genotype*stress effect ^#^*P* < 0.05; ^##^*P* < 0.01; ^###^*P* < 0.001; wt = wild-type mice; BL = baseline; wt n = 16; (*Casp1, Ifngr, Nos2*)^−/−^ n = 20.

In the open field test, a significant stress-genotype interaction was observed for total distance travelled (F_1,34_ = 11.091, *P* = 0.002) (Fig. 3a). This result was driven by the 17.5% decrease in locomotor activity in (*Casp1, Ifngr, Nos2*)^−/−^ mice following stress (F_1,34_ = 24.68, *P* < 0.001), while the distance travelled by wt mice was unchanged (F_1,34_ = 0, *P* = 0.981) compared to baseline. Similarly, a significant stress-genotype interaction was observed for the average moving velocity in the open field test (F_1,34_ = 11.154, *P* = 0.002) (Fig. 3b). While the average velocity recorded for wt mice was unchanged (F_1,34_ = 0, *P* = 0.979) following CUS, (*Casp1, Ifngr, Nos2*)^−/−^ mice displayed a statistically significant 17.6% reduction (F_1,34_ = 24.80, *P* < 0.001). A significant stress-genotype interaction was recorded for the number of faecal boli in the open field test (F_1,34_ = 14.285, *P* < 0.001) (Fig. 2c). While this measure did not differ significantly between genotypes at baseline (F_1,34_ = 2.11, *P* = 0.155), and was unchanged in wt mice following chronic stress (F_1,34_ = 0.58, *P* = 0.453), it was increased in (*Casp1, Ifngr, Nos2*)^−/−^ mice following stress (68.4% increase, F_1,34_ = 23.23, *P* < 0.001).

### *Casp1*, *Ifngr* and *Nos2* deficiency increases locomotor activity

*Casp1*, *Nos2* and *Ifngr* deficiency was associated with increased locomotor activity (F_1,34_ = 58.883, *P* < 0.001; Fig. 3a) and average moving velocity (F_1,34_ = 58.777, *P* < 0.001; Fig. 3b) in the open field test in comparison to wt mice. Furthermore, the number of centre visits but not the total time (Fig. 2b) spent in the centre was increased in (*Casp1, Ifngr, Nos2*)^−/−^ mice (respectively F_1,34_ = 35.424, *P* < 0.001 and F_1,34_ = 0.200, *P* = 0.658) in the open field test. Moreover, (*Casp1, Ifngr, Nos2*)^−/−^ mice displayed increased number of entries in the closed arms of the elevated plus maze (F_1,34_ = 64.426, *P* < 0.001) and in the total number of entries in any of the arms, irrespective of whether they were open or closed (F_1,34_ = 20.348, *P* < 0.001) (Fig. 3c).

### *Casp1*, *Ifngr* and *Nos2* deficiency does not affect ACTH and CORT plasma levels following stress

**Figure 4:**
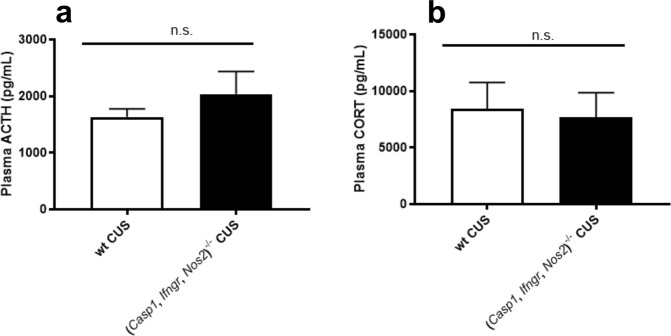
*Casp1*, *Ifngr* and *Nos2* deficiency does not affect the levels of adrenocorticotropic hormone and corticosterone following chronic unpredictable stress. (**a**) (*Casp1, Ifngr, Nos2*)^−/−^ mice displayed similar levels of adrenocorticotropic hormone (ACTH), and (**b**) corticosterone (CORT) compared to wild-type (wt) mice at euthanasia. Data are presented as means ± s.e.m. n.s. = not significant. ACTH wt n = 14, *(Casp1, Ifngr, Nos2*)^−/−^ n = 13; CORT wt n = 12, *(Casp1, Ifngr, Nos2*)^−/−^ n = 18.

At the endpoint of the experiment, *Casp1, Ifngr* and *Nos2* deficiency was not associated with altered plasma levels of ACTH (t_2,25_ = 0.1465, *P* = 0.8847) (Fig. 4a) or CORT (t_2,28_ = 0.3851, *P* = 0.7031) (Fig. 4b) when compared to wt mice.

### CUS affects anxiety- and depressive-like behaviour

28-day CUS exposure exacerbated depressive-like behaviour, as measured by increased floating (F_1,34_ = 4.299, *P* = 0.046; Fig. 1a) and decreased climbing behaviours in the forced swim test (F_1,34_ = 6.545, *P* = 0.015; Fig. 1c). Moreover, CUS induced anhedonia, as measured by a decreased preference for a 1% sucrose solution in the sucrose preference test (F_1,34_ = 50.384, *P* < 0.001; Fig. 1d). Furthermore, CUS increased anxiety-like behaviour, as it decreased the time spent in the open arms of the elevated plus maze (F_1,34_ = 10.423, *P* = 0.003; Fig. 2a), decreased the open/closed arms entries ratio (F_1,34_ = 15.595, *P* < 0.001) and decreased the time spent in the centre section of the open field test arena (F_1,34_ = 12.583, *P* < 0.001; Fig. 2b). However, no differences were observed in the centre distance/total distance ratio in the open field test (F_1,34_ = 2.442, *P* = 0.127).

### *Casp1*, *Ifngr* and *Nos2* deficiency affects gut microbiome composition

To determine whether *Casp1, Ifngr* and *Nos2* deficiency influenced the gut microbiota composition, faecal samples were analysed by 16S rRNA sequencing (Supplementary Fig. 2). Alpha diversity analysis indicated that the faecal microbial community of (*Casp1, Ifngr, Nos2*)^−/−^ mice had significantly increased richness (number of bacterial taxa observed), evenness and diversity when compared to wt (*P* < 0.0001) (Supplementary Fig. 3). Besides, the faecal microbiota composition of wt and (*Casp1, Ifngr, Nos2*)^−/−^ mice differed significantly, as determined by permutational ANOVA (PERMANOVA) analysis (*P*(perm) = 0.0001, Pseudo-F = 38.22, 9950 permutations).

**Figure 5:**
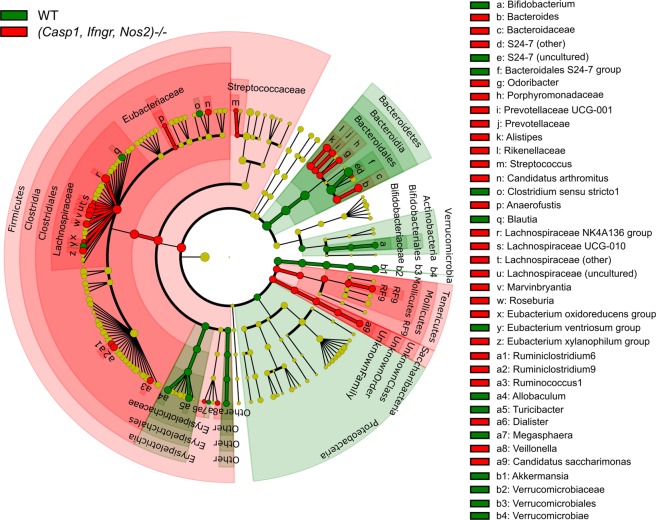
Taxonomic cladogram based on LEfSe analysis of bacterial taxa identified by 16S rRNA sequencing of faecal samples. Taxa that are enriched in wt and (*Casp1, Ifngr, Nos2*)^−/−^ faecal samples are indicated in green and red, respectively. Differential taxa were determined based on an LDA threshold score of >3 and a statistical significance level of 0.05.

Comparison of genus level relative abundances between the groups using LEfSe analysis indicated that the relative abundance of several genera within the Lachnospiraceae family including *Anaerofustis, Marvinbryantia,* and *Roseburia*, as well as *Ruminicococcus* and *Ruminiclostridium*, *Streptococcus, Dialister*, and *Veillonella* were significantly higher in (*Casp1, Ifngr, Nos2*)^−/−^ mice (Fig. 5). Significantly higher relative abundances were also observed for other genera, including *Bacteroides, Odoribacter, Prevotellaceae UCG-001*, and *Alistipes* in (*Casp1, Ifngr, Nos2*)^−/−^ mice. The relative abundance of S24-7 (a member of the order Bacteroidales) was significantly lower in (*Casp1, Ifngr, Nos2*)^−/−^ mice. Also, the relative abundance of *Bifidobacterium, Allobaculum, Turicibacter*, and the mucin-degrading bacterium *Akkermansia* were significantly lower in (*Casp1, Ifngr, Nos2*)^−/−^ mice.

### *Casp1*, *Ifngr* and *Nos2* deficiency affects the change in gut microbiota composition in response to chronic stress

Assessment of the faecal microbial community at baseline and after 28 days of CUS treatment showed no significant changes in measures of richness, evenness and diversity in (*Casp1, Ifngr, Nos2*)^−/−^ mice following stress (*P* > 0.05) (Supplementary Fig. 4). Significant increases in microbial evenness were observed for wt mice following stress (*P* = 0.004), but not for (*Casp1, Ifngr, Nos2*)^−/−^ mice, when compared to their respective baselines. Microbial richness or diversity did not differ in either genotype when subject to CUS (*P* > 0.005). However, stochastic changes for microbial richness, which include significant increases in the wt control group and a significant decrease in the (*Casp1, Ifngr, Nos2*)^−/−^ control group were observed. PERMANOVA analysis indicated a significant interaction between genotype, time and treatment on the faecal microbiota composition (*P*(perm) = 0.017, Pseudo-F = 2.978, 9939 permutations).

**Figure 6:**
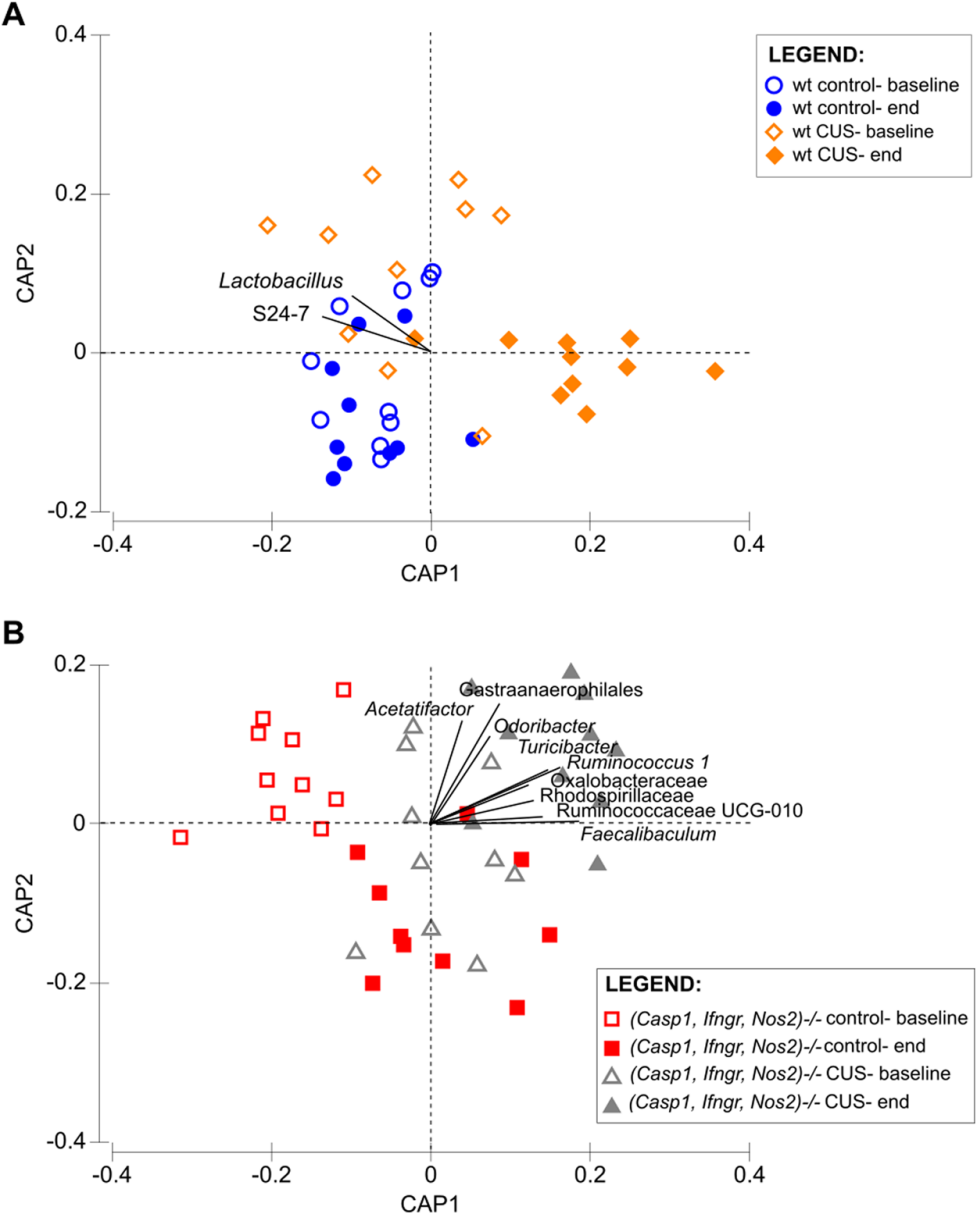
Canonical analysis of principal coordinates (CAP) biplot constrained by time and treatment. The biplot was determined based on a Spearman’s correlation score of 0.4. Only bacterial taxa in the two opposing quadrants separating the CUS (chronic unpredictable stress) group pre- and post-treatment are indicated.

To determine the influence of stress on faecal microbiota, pairwise comparisons between pre- and post-treatment samples were performed for all groups. CUS for 28 days significantly altered the faecal microbiota of wt mice (*P*(perm) = 0.0012, t = 3.213, 9931 permutations). Significant compositional changes were also observed in control (*Casp1, Ifngr, Nos2*)^−/−^ (*P*(perm) = 0.004, t = 3.049, 9929 permutations) and stressed (*Casp1, Ifngr, Nos2*)^−/−^ mice (*P*(perm) = 0.010, t = 1.739, 9915 permutations). Bacterial taxa that were altered in wt or (*Casp1, Ifngr, Nos2*)^−/−^ mice as a result of CUS were identified by CAP analysis. Based on CAP biplots, bacterial taxa in the opposing quadrants separating the pre- and post-chronic stress groups for the wt (Fig. 6A) or (*Casp1, Ifngr, Nos2*)^−/−^ mice (Fig. 6B) were identified for further statistical analysis. The bacterial taxa *Lactobacillus* and S24-7 were found to contribute substantially to the compositional differences observed in wt mice following stress, while the taxa *Acetatifactor, Odoribacter, Turicibacter, Ruminococcus1, Faecalibaculum*, Gastranaerophilales, and Ruminococcaceae UCG-010 were found to contribute to the differences in the (*Casp1, Ifngr, Nos2*)^−/−^ genotype following stress exposure. While the taxon *Bifidobacterium* was not identified by CAP analysis, further comparisons were also performed for this taxon due to its documented contribution to the stress response^22,23^.

**Figure 7:**
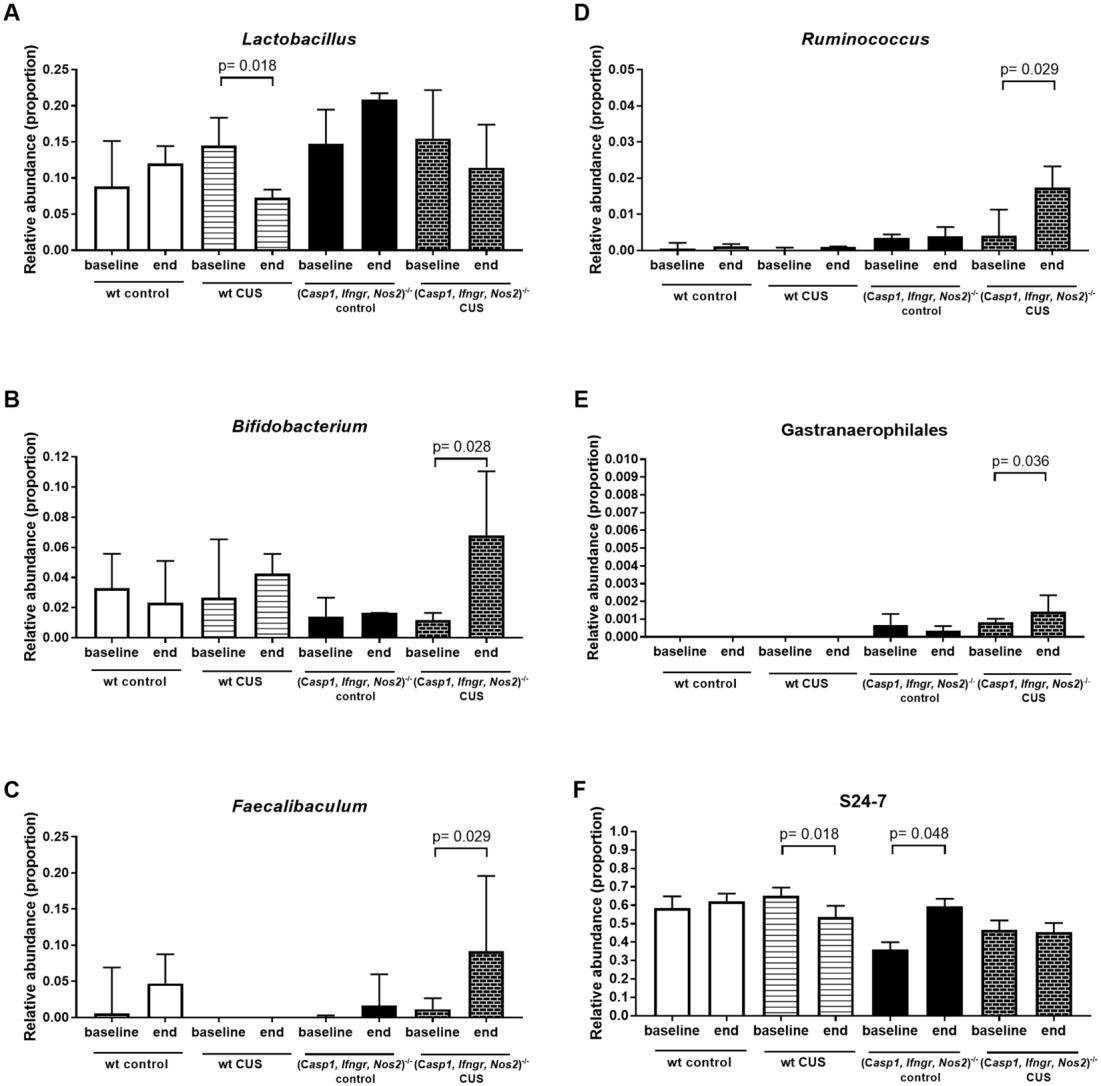
Relative abundance of selected bacterial taxa that were identified to be differential due to chronic stress. Identification of bacterial taxa was based on the CAP biplots. Pairwise comparison between time-points was performed for each group using the Wilcoxon test based on a statistical significance level of 0.05. CUS, chronic unpredictable stress.

Pairwise comparison of the pre- and post-treatment relative abundances of bacterial taxa confirmed a significant reduction in the relative abundance of *Lactobacillus* in wt mice following stress (FDR *P* = 0.018), but not in (*Casp1, Ifngr, Nos2*)^−/−^ mice (Fig. 7A). An increased relative abundance of *Bifidobacterium* (FDR *P* = 0.028) (Fig. 7B), *Faecalibaculum* (FDR *P* = 0.029) (Fig. 7C), *Ruminococcus1* (FDR *P* = 0.029) (Fig. 7D) and Gastranaerophilales (FDR *P* = 0.036) (Fig. 7E) were observed in (*Casp1, Ifngr, Nos2*)^−/−^ mice subjected to CUS, and not in the control groups. Significant decreases in the relative abundance of S24-7 were observed in wt CUS mice (FDR *P* = 0.018) but not in (*Casp1, Ifngr, Nos2*)^−/−^ CUS mice, although increased relative abundance was observed in (*Casp1, Ifngr, Nos2*)^−/−^ control mice (FDR *P* = 0.049) (Fig. 7F). Ruminococcaceae UCG-010 (FDR *P* = 0.029) and *Turicibacter* (FDR *P* = 0.029) relative abundances significantly increased in the (*Casp1, Ifngr, Nos2*)^−/−^ CUS group and the control groups, independent of genotype [(*Casp1, Ifngr, Nos2*)^−/−^ (FDR *P* = 0.048) and wt (FDR *P* = 0.018)].

## Discussion

Increasing evidence suggests a dysregulation of neuroinflammatory pathways and a potential alteration of gut microbiome equilibrium in MDD. We aimed to determine whether genetic deletion of *Casp1*, *Nos2* and *Ifngr* affects depression- and anxiety-like behaviours in mice, either in the absence of stress or in response to CUS. Furthermore, we also determined whether faecal microbiota composition was changed as a result of altered genotype, either in isolation or when combined with chronic stress.

Mice deficient in *Casp1, Ifngr* and *Nos2* displayed decreased depressive-like behaviour at baseline, as measured by decreased floating time in the forced swim test, and increased hedonic-like behaviour, as measured by increased sucrose preference. Moreover, (*Casp1, Ifngr, Nos2*)^−/−^ mice displayed decreased levels of anxiety-like behaviour in the elevated plus maze, as they spent more time in the open arms of the maze compared to wt mice. These mice also exhibited increased locomotor activity and moving velocity.

After 28 days of CUS exposure, we observed a different stress response in *(Casp1, Ifngr, Nos2)*^−/−^ mice. CUS increased floating and decreased swimming and struggling time, as reported previously^24,25^. While CUS decreased sucrose preference in both wt and (*Casp1, Ifngr, Nos2*)^−/−^ genotypes, the decrease observed in wt mice was greater and fell below the anhedonic threshold of 65% preference, while the decrease in (*Casp1, Ifngr, Nos2*)^−/−^ mice did not, suggesting that (*Casp1, Ifngr, Nos2*)^−/−^ mice are protected from developing anhedonic-like behaviour following stress. In accordance with this finding, inhibition of hippocampal NOS2 was shown to prevent the decrease in sucrose preference following CUS^26^. Similarly, NOS2 inhibition results in antidepressant-like effects in mice while preventing the exacerbation of depressive-like behaviour^11^. Accordingly, it was reported that *Ifng* deficiency affects basal emotionality while blunting some of the behavioural and biochemical responses to chronic stressors^12^. *Casp1*^−/−^ mice have decreased basal anxiety and depressive-like behaviour and decreased exacerbation of those behaviours following stress exposure^27^. Simultaneously inhibiting *Casp1, Ifngr* and *Nos2* may result in a complex neuro-behavioural phenotype, different from individual gene knockouts, which decreases anxiety- and depressive-like behaviours at baseline, while preventing the exacerbation of anhedonic- but not of depressive- or anxiety-like behaviours following chronic stress exposure.

(*Casp1, Ifngr, Nos2*)^−/−^ mice, but not wt mice, displayed a higher number of faecal pellets excreted during the open field test following stress exposure. Similar effects have been reported in germ-free mice and rats exposed to stress^28,29^. It has been hypothesised that such increases result from stress-induced colonic hypermotility and hyperalgesia^28,29^. This finding is in contrast to a previous report that wt, but not *Casp1*^−/−^ mice, displayed an increased number of defecations in the open field test following chronic restraint stress, which was hypothesized to reflect stress resilience in *Casp1*^−/−^ mice^27^. These apparently contrasting findings might be due to the different stress paradigms used in the present study and/or to the different gut microbiome composition of the (*Casp1, Ifngr, Nos2*)^−/−^ genotype. Although studying a causal correlation between faecal output and gut microbiome composition was beyond the scope of this study, it cannot be excluded that changes in faecal output might be mediated by the observed changes in gut bacteria composition between genotypes.

There are many potential explanations for the increased locomotor activity displayed by (*Casp1, Ifngr, Nos2*)^−/−^ mice. Pre-clinical studies have identified several systems that modulate spontaneous locomotor activity such as the dopaminergic system, the glutamatergic system, and the caspase 1 system^27,30–33^. Hence, it could be hypothesized that (*Casp1, Ifngr, Nos2*)^−/−^ mice have increased levels of movement-related neurotransmitters, such as dopamine or glutamate. In order to test this hypothesis, future studies might interrogate monoamines levels in brain regions that are relevant to locomotion (e.g., substantia nigra and striatum) and MDD (e.g., prefrontal cortex and hippocampus) in the transgenic model used in this study. If (*Casp1, Ifngr, Nos2*)^−/−^ mice indeed have increased levels of dopamine and/or glutamate, they might prove useful as pre-clinical models of Parkinson’s disease. In this disease, the main neurodegenerative features include inflammation-mediated loss of dopaminergic neurons in areas involved with movement and locomotor activity, such as the substantia nigra and the striatum^34^. Unsurprisingly, it has been shown that the CASP1 and CASP3 inhibitor minocycline prevents nigrostriatal dopaminergic neurodegeneration in a mouse model of Parkinson’s disease^35^.

Faecal microbiota analysis indicated significant compositional differences between (*Casp1, Ifngr, Nos2*)^−/−^ and wt mice. Notably, rather than affecting the gut microbiota as a whole, the deletion of *Casp1, Ifngr* and *Nos2* genes result in specific and reproducible changes in the relative abundance of discrete bacterial taxa. Previous studies have shown the effect of *Casp1*^−/−^ gene knockout on gut microbiota composition^36,37^. These studies indicated an overrepresentation of Prevotellaceae in *Casp1*^−/−^ mice, which were recapitulated in the (*Casp1, Ifngr, Nos2*)^−/−^ genotype in this study. However, in contrast to our findings, the *Casp1*^−/−^ mice described by Brinkman and colleagues exhibited decreased Lachnospiraceae relative abundance, while another study showed that *Ifng* and *Ifngr*-deficient mice had decreased abundance of *Bacteroides/Prevotella* or bifidobacteria^37,38^. The discrepancy in taxa relative abundances could be due to the effect on gut microbiome composition of multiple, as opposed to single, gene deletions. Previous studies have shown that deletion of genes involved in immune function and exposure to opportunistic pathogens can influence commensal microbiota composition^39^.

Exposure to CUS for 28 days resulted in a significant decrease in the relative abundance of *Lactobacillus* in wt mice, compared to baseline levels, consistent with trends demonstrated in other CUS studies^40^ and pre-clinical models of social stress^41,42^. Accordingly, decreased *Lactobacillus* levels have been reported in MDD patients^43^. Interestingly, *Lactobacillus* levels did not decrease in (*Casp1, Ifngr, Nos2*)^−/−^ mice after CUS, suggesting that their genetic makeup may provide resistance towards *Lactobacillus* shifts and that this trait might be involved in their observed resilience to developing anhedonic-like behaviour. *Lactobacillus* has been suggested to have beneficial effects such as reducing levels of TNFA and IFNG in human intestinal epithelial cells^44^, and those cytokines are associated with MDD^45^ and pre-clinical MDD models^20^. Indeed, monocolonisation of germ-free mice with *Lactobacillus plantarum* PS128 improves anxiety-like behaviour and increases striatal serotonin and dopamine levels^20^, while chronic treatment with *Lactobacillus rhamnosus* reduces corticosterone levels in a vagal-dependent manner^46^.

The relative abundance of *Bifidobacterium, Faecalibaculum* and *Ruminococcus* significantly increased in (*Casp1, Ifngr, Nos2*)^−/−^ mice subjected to CUS treatment but not in wt CUS mice or those in the control treatment groups. Our findings are consistent with our previous study, which showed that mice subjected to chronic restraint stress had reduced levels of *Bifidobacterium*^27^. Increased *Bifidobacterium* levels are associated with resilience to chronic social defeat stress, while *Bifidobacterium* supplementation increases resilience status in previously susceptible mice^23^. Monocolonisation of germ-free mice with *Bifidobacterium infantis* reverses the exaggerated HPA stress response observed in these mice, potentially by preventing the elevation of stress hormones such as ACTH and CORT^22^.

*Ruminococcus* and *Bifidobacterium*, which were increased in (*Casp1, Ifngr, Nos2*)^−/−^ mice following stress, are both able to produce short chain fatty acids (SCFA)^47^. Similarly, *Faecalibaculum* was also increased and can produce lactic acid, a precursor of the SCFA butyrate^48^. Given that SCFA may play a role in reducing stress responses by reducing intestinal epithelial barrier permeability^17^, it cannot be excluded that the increase of those taxa in (*Casp1, Ifngr, Nos2*)^−/−^ mice following stress might be involved in their resilience to developing anhedonic-like behaviour.

## Limitations of the Study

The mouse model used in this study simultaneously lacks three genes, making the assignment of observed effects to particular proteins difficult. The rationale to generate this model stemmed from the evidence that each single knockout of these genes displays altered responses to stress or antidepressant-like phenotypes^10–12^; therefore, we hypothesized that by combining these knockouts, the effects might be additive and result in a greater antidepressant-like phenotype. In order to bypass this limitation, it might prove valuable to compare the present results with single KO mouse models for each of the genes investigated, while generating combinations of double KOs to ascertain if they have greater antidepressant-like behaviour than the respective individual KOs. Another limitation of this study is that, although the circulating levels of adrenocorticotropic hormone and corticosterone were measured following CUS, such measurements were not performed at the beginning of the stress regime. Further studies should determine whether (*Casp1, Ifngr, Nos2*)^−/−^ mice present altered levels of ACTH and/or CORT at baseline. Lastly, the changes in gut microbiome observed between genotypes and stress groups are correlative nature, rather than causal.

## Conclusions

In conclusion, we investigated the effects of simultaneous *Casp1*, *Ifngr* and *Nos2* gene deletion in a CUS model of stress-induced depressive-like behaviour. (*Casp1, Ifngr, Nos2*)^−/−^ mice displayed decreased levels of depressive- and anxiety-like behaviour while exhibiting increased locomotor activity and moving velocity. Following chronic stress exposure, (*Casp1, Ifngr, Nos2*)^−/−^ mice present an attenuated anhedonic-like behaviour compared to wt mice while presenting no alterations in ACTH and CORT plasma levels compared to wt at the time of euthanasia. Correlative faecal microbiota changes were observed as a function of genotype, stress exposure, and genotype-stress interaction. Future studies are needed to investigate the clinical potential of inhibiting these pro-inflammatory signalling pathways in MDD.

## Methods

All procedures were approved by the South Australian Health and Medical Research Institute (SAHMRI) Ethics committee and are in accordance with the Australian Code for the Care and Use of Animals for Scientific Purposes (8^th^ edition, 2013). Male C57BL/6 J mice (wild-type, wt, n = 26) aged 60 days were obtained from the SAHMRI Bioresources Facility (Adelaide, Australia). Age-matched (*Casp1, Ifngr, Nos2*)^−/−^ mice (n = 30) with C57BL/6 J background were generated by back-crossing mice with individual gene deletions^49–51^.

### Chronic unpredictable stress

The CUS protocol consisted of 28 days exposure to randomly scheduled, low and mild intensity social and environmental stressors, applied each day during the light phase of the light cycle. Briefly, the stressors were: (a) restraint, (b) removal of bedding and nesting material, (c) soiled bedding, (d) 45° cage tilting, (e) predator stress, (f) forced swim test, (g) fasting, (h) social stress, (i) light cycle disruption and (j) light cycle reversal (for detailed procedures refer to Supplementary Table 2 and Supplementary Methods).

### Behavioural testing

Mice were submitted to the open field, elevated plus maze, forced swim and sucrose preference tests at baseline and following CUS. All tests were video-recorded and analysed using Ethovision XT 10 software (Noldus, Wageningen, Holland) for behaviour recognition and scoring. See Supplementary Fig. 1 for a timeline of experimental procedures.

### Adrenocorticotropic hormone (ACTH) and corticosterone (CORT) measurements

Plasma ACTH and plasma CORT level were measured respectively by a competitive inhibition ELISA kit (Cloud-Clone Corp., Wuhan, Hubei, China) and a competitive immunoassay ELISA kit (Enzo Life Sciences, Farmingdale, New York, USA) following the manufacturers’ instructions.

### 16S rRNA gene amplicon sequencing and bioinformatics analysis

Fresh faecal pellets were collected between 10–11 am with sterile toothpicks on experimental day 41 during weighing procedures and placed into sterile 1.5 mL Eppendorf tubes and stored at −80 °C. DNA was extracted from faecal samples using the DNeasy PowerSoil HTP 96 kit (Qiagen, Hilden, Germany) optimized for the Biomek 4000 Automation Workstation (Beckman Coulter Inc., Lane Cove, NSW, Australia). Barcoded amplicons of the V4 hypervariable region of the bacterial 16S rRNA gene were generated from the faecal DNA extracts based on the Illumina Miseq. 16S Metagenomic Sequencing Library Preparation protocol with modifications, as described previously^52^, and sequencing was performed on an Illumina Miseq platform. A detailed description of library preparation and sequencing are provided in the Supplementary Methods.

Bioinformatics analysis of paired-end sequences was performed using Quantitative Insights Into Microbial Ecology (QIIME, v1.9.1)^53^ based on a previously described bioinformatics pipeline^54^. Paired forward and reverse barcoded reads were quality filtered and merged using the Paired-End read merger (PEAR v0.9.10)^55^. Chimera filtering (USEARCH, v6.1)^56^ and open reference operational taxonomic unit (OTU) assignment was performed against the SILVA reference database (release 128) that were clustered at 97% identity. Only sequences with >80% identity to the reference sequence were used for OTU assignment step. All samples had spurious OTUs removed and were subsampled to the lowest sequence depth at 7,354 sequence reads, providing an average Good’s coverage score of 97.13% (±0.7%).

### Statistical analysis

Power analysis was performed based on the effect size seen in a previous pilot study investigating the effects of simultaneous *Casp1, Ifngr and Nos2* deficiency on total floating time in the forced swim test (our primary outcome measure). Statistical analyses of the behavioural tests were performed using the Statistical Package for the Social Sciences version 23.0 for Windows (SPSS, Chicago, Illinois) using a general linear model for repeated measures (repeated measures ANOVA). The effects of genotype, stress, treatment and their interaction were explored and the significance set at *P* ≤ 0.05. Statistically significant stress-genotype interactions were further assessed as described previously^57^. ELISA data were analysed by two-tailed unpaired student T-test. Alpha diversity scores of observed species, Simpson (1-D) and Shannon were calculated using QIIME^53^. Beta-diversity analyses were performed using the PRIMER software (v6, PRIMER-E Ltd, Plymouth, UK). Comparison of microbiota composition between groups was performed on weighted Unifrac distances using a permutational multivariate analysis of variance (PERMANOVA) test with 9,999 permutations. LEfSe (linear discriminant analysis effect size) analysis^58^ was used for the comparison of bacterial taxa relative abundances between genotypes, based on a Linear Discriminant Analysis (LDA) threshold score of 3. Specific taxa that contributed to group differences in the wt or (*Casp1, Ifngr and Nos2*)^−/−^ group following stress were identified using a canonical analysis of principal coordinates (CAP) plot generated based on the factors treatment (control, CUS) and time (baseline, 28 days). Within-group paired comparisons of the relative abundances of specific taxa were further assessed using the Wilcoxon test with a false discovery rate (FDR) correction for multiple testing. The significance level was set at 0.05 for all statistical tests.

## Supplementary information

Supplementary Material

## References

[1] Leonard B, Maes M (2012). Mechanistic explanations how cell-mediated immune activation, inflammation and oxidative and nitrosative stress pathways and their sequels and concomitants play a role in the pathophysiology of unipolar depression. Neurosci Biobehav Rev.

[2] Licinio J, Wong ML (1999). The role of inflammatory mediators in the biology of major depression: central nervous system cytokines modulate the biological substrate of depressive symptoms, regulate stress-responsive systems, and contribute to neurotoxicity and neuroprotection. Mol Psychiatry.

[3] Black CN, Bot M, Scheffer PG, Cuijpers P, Penninx BW (2015). Is depression associated with increased oxidative stress? A systematic review and meta-analysis. Psychoneuroendocrinology.

[4] Kudlow P, Cha DS, Carvalho AF, McIntyre RS (2016). Nitric Oxide and Major Depressive Disorder: Pathophysiology and Treatment Implications. Curr Mol Med.

[5] Alcocer-Gomez E (2014). NLRP3 inflammasome is activated in mononuclear blood cells from patients with major depressive disorder. Brain Behav Immun.

[6] Kaufmann FN (2017). NLRP3 inflammasome-driven pathways in depression: Clinical and preclinical findings. Brain Behav Immun.

[7] Maes M (1994). Increased neopterin and interferon-gamma secretion and lower availability of L-tryptophan in major depression: further evidence for an immune response. Psychiatry Res.

[8] Myint AM, Leonard BE, Steinbusch HW, Kim YK (2005). Th1, Th2, and Th3 cytokine alterations in major depression. J Affect Disord.

[9] Inserra, A., Mastronardi, C. A., Rogers, G., Licinio, J. & Wong, M. L. Neuroimmunomodulation in Major Depressive Disorder: Focus on Caspase 1, Inducible Nitric Oxide Synthase, and Interferon-Gamma. Mol Neurobiol, 10.1007/s12035-018-1359-3 (2018). 10.1007/s12035-018-1359-3 PMC6505498 30306457

[10] Wong ML (2016). Inflammasome signaling affects anxiety- and depressive-like behavior and gut microbiome composition. Mol Psychiatry.

[11] Montezuma K (2012). Inhibition of iNOS induces antidepressant-like effects in mice: pharmacological and genetic evidence. Neuropharmacology.

[12] Litteljohn D (2010). Interferon-gamma deficiency modifies the effects of a chronic stressor in mice: Implications for psychological pathology. Brain Behav Immun.

[13] Peterson CT, Sharma V, Elmen L, Peterson SN (2015). Immune homeostasis, dysbiosis and therapeutic modulation of the gut microbiota. Clin Exp Immunol.

[14] Wang Y, Kasper LH (2014). The role of microbiome in central nervous system disorders. Brain Behav Immun.

[15] Rogers GB (2016). From gut dysbiosis to altered brain function and mental illness: mechanisms and pathways. Mol Psychiatry.

[16] Sudo N, Microbiome HPA (2014). axis and production of endocrine hormones in the gut. Adv Exp Med Biol.

[17] Carabotti M, Scirocco A, Maselli MA, Severi C (2015). The gut-brain axis: interactions between enteric microbiota, central and enteric nervous systems. Ann Gastroenterol.

[18] Zheng P (2016). Gut microbiome remodeling induces depressive-like behaviors through a pathway mediated by the host’s metabolism. Mol Psychiatry.

[19] Chen JJ (2018). Sex differences in gut microbiota in patients with major depressive disorder. Neuropsychiatr Dis Treat.

[20] Liu YN (2015). TNFalpha mediates stress-induced depression by upregulating indoleamine 2,3-dioxygenase in a mouse model of unpredictable chronic mild stress. Eur Cytokine Netw.

[21] Inserra A, Rogers GB, Licinio J, Wong ML (2018). The Microbiota-Inflammasome Hypothesis of Major Depression. Bioessays.

[22] Sudo N (2004). Postnatal microbial colonization programs the hypothalamic-pituitary-adrenal system for stress response in mice. J Physiol.

[23] Yang C (2017). Bifidobacterium in the gut microbiota confer resilience to chronic social defeat stress in mice. Sci Rep.

[24] Zhu S, Shi R, Wang J, Wang JF, Li XM (2014). Unpredictable chronic mild stress not chronic restraint stress induces depressive behaviours in mice. Neuroreport.

[25] Zhu S (2014). Unpredictable chronic mild stress induces anxiety and depression-like behaviors and inactivates AMP-activated protein kinase in mice. Brain Res.

[26] Wang D, An SC, Zhang X (2008). Prevention of chronic stress-induced depression-like behavior by inducible nitric oxide inhibitor. Neurosci Lett.

[27] Wong M-L, Inserra A, Lewis M D, Mastronardi C A, Leong L, Choo J, Kentish S, Xie P, Morrison M, Wesselingh S L, Rogers G B, Licinio J (2016). Inflammasome signaling affects anxiety- and depressive-like behavior and gut microbiome composition. Molecular Psychiatry.

[28] De Palma G (2015). Microbiota and host determinants of behavioural phenotype in maternally separated mice. Nat Commun.

[29] Bradesi S (2005). Repeated exposure to water avoidance stress in rats: a new model for sustained visceral hyperalgesia. Am J Physiol Gastrointest Liver Physiol.

[30] Gainetdinov RR, Mohn AR, Bohn LM, Caron MG (2001). Glutamatergic modulation of hyperactivity in mice lacking the dopamine transporter. Proc Natl Acad Sci USA.

[31] Burns LH, Everitt BJ, Kelley AE, Robbins TW (1994). Glutamate-dopamine interactions in the ventral striatum: role in locomotor activity and responding with conditioned reinforcement. Psychopharmacology (Berl).

[32] Swanson CJ, Kalivas PW (2000). Regulation of locomotor activity by metabotropic glutamate receptors in the nucleus accumbens and ventral tegmental area. J Pharmacol Exp Ther.

[33] Karlsson RM, Tanaka K, Heilig M, Holmes A (2008). Loss of glial glutamate and aspartate transporter (excitatory amino acid transporter 1) causes locomotor hyperactivity and exaggerated responses to psychotomimetics: rescue by haloperidol and metabotropic glutamate 2/3 agonist. Biol Psychiatry.

[34] McGeer PL, McGeer EG (2004). Inflammation and neurodegeneration in Parkinson’s disease. Parkinsonism Relat Disord.

[35] Du Y (2001). Minocycline prevents nigrostriatal dopaminergic neurodegeneration in the MPTP model of Parkinson’s disease. Proc Natl Acad Sci USA.

[36] Elinav E (2011). NLRP6 inflammasome regulates colonic microbial ecology and risk for colitis. Cell.

[37] Brinkman BM (2011). Caspase deficiency alters the murine gut microbiome. Cell Death Dis.

[38] Thoene-Reineke C (2014). Composition of intestinal microbiota in immune-deficient mice kept in three different housing conditions. PLoS One.

[39] Galvez EJC, Iljazovic A, Gronow A, Flavell R, Strowig T (2017). Shaping of Intestinal Microbiota in Nlrp6- and Rag2-Deficient Mice Depends on Community Structure. Cell Rep.

[40] Marin IA (2017). Microbiota alteration is associated with the development of stress-induced despair behavior. Sci Rep.

[41] Bailey MT (2011). Exposure to a social stressor alters the structure of the intestinal microbiota: implications for stressor-induced immunomodulation. Brain Behav Immun.

[42] Galley JD (2014). Exposure to a social stressor disrupts the community structure of the colonic mucosa-associated microbiota. BMC Microbiol.

[43] Aizawa E (2016). Possible association of Bifidobacterium and Lactobacillus in the gut microbiota of patients with major depressive disorder. J Affect Disord.

[44] Resta-Lenert S, Barrett KE (2006). Probiotics and commensals reverse TNF-alpha- and IFN-gamma-induced dysfunction in human intestinal epithelial cells. Gastroenterology.

[45] Mikova O, Yakimova R, Bosmans E, Kenis G, Maes M (2001). Increased serum tumor necrosis factor alpha concentrations in major depression and multiple sclerosis. Eur Neuropsychopharmacol.

[46] Bravo JA (2011). Ingestion of Lactobacillus strain regulates emotional behavior and central GABA receptor expression in a mouse via the vagus nerve. Proc Natl Acad Sci USA.

[47] LeBlanc JG (2017). Beneficial effects on host energy metabolism of short-chain fatty acids and vitamins produced by commensal and probiotic bacteria. Microb Cell Fact.

[48] Lim S, Chang DH, Ahn S, Kim BC (2016). Whole genome sequencing of “Faecalibaculum rodentium” ALO17, isolated from C57BL/6J laboratory mouse feces. Gut Pathog.

[49] Kuida K (1995). Altered cytokine export and apoptosis in mice deficient in interleukin-1 beta converting enzyme. Science.

[50] Laubach VE, Shesely EG, Smithies O, Sherman PA (1995). Mice lacking inducible nitric oxide synthase are not resistant to lipopolysaccharide-induced death. Proc Natl Acad Sci USA.

[51] Huang S (1993). Immune response in mice that lack the interferon-gamma receptor. Science.

[52] Choo JM, Leong LE, Rogers GB (2015). Sample storage conditions significantly influence faecal microbiome profiles. Sci Rep.

[53] Caporaso JG (2010). QIIME allows analysis of high-throughput community sequencing data. Nat Methods.

[54] Jervis-Bardy J (2015). Deriving accurate microbiota profiles from human samples with low bacterial content through post-sequencing processing of Illumina MiSeq data. Microbiome.

[55] Zhang J, Kobert K, Flouri T, Stamatakis A (2014). PEAR: a fast and accurate Illumina Paired-End reAd mergeR. Bioinformatics.

[56] Edgar RC (2010). Search and clustering orders of magnitude faster than BLAST. Bioinformatics.

[57] Kinnear, P. R. & Gray, C. D. PASW statistics 17 made simple (replaces SPSS statistics 17). 1st edn, (Psychology Press, 2010).

[58] Segata N (2011). Metagenomic biomarker discovery and explanation. Genome Biol.

