# Supplementary material for: Mice lacking Casp1, Ifngr and Nos2 genes exhibit altered depressive- and anxiety-like behaviour, and gut microbiome composition
Source: Sci Rep. 2019 Apr 23;9:6456. doi: 10.1038/s41598-018-38055-8 (PMC6478737; doi:10.1038/s41598-018-38055-8)
Supplement: Supplementary file 1 — Supplementary Material [file 41598_2018_38055_MOESM1_ESM.pdf]

# SUPPLEMENTARY MATERIAL

# Mice lacking *Casp1*, *Ifngr* and *Nos2* genes exhibit altered depressive- and anxiety-like behaviour, and gut microbiome composition

**Authors**: Antonio Inserra [a, b, c] *, Jocelyn Choo [d, e] *, Martin D Lewis [a, b], Geraint B Rogers [d, e] #, Ma-Li Wong [a, b, f #] and Julio Licinio [a, b, f #].

*** Both authors contributed equally to this work.**

**# These authors are co-contributing senior authors.**

**Affiliation:** [a] Mind and Brain Theme, South Australian Health and Medical Research Institute, Adelaide, SA, Australia; [b] Discipline of Psychiatry, College of Medicine and Public Health, Flinders University, Bedford Park, SA, Australia; [c] Centre for Neuroscience, Flinders University, Bedford Park, Australia; [d] Infection and Immunity Theme, South Australia Health and Medical Research Institute, North Terrace, Adelaide, SA, Australia; [e] SAHMRI Microbiome Research Laboratory, Flinders University College of Medicine and Public Health, Bedford Park, SA, Australia; [f] State University of New York Upstate Medical University, Syracuse, NY, USA.

**Correspondence:** Professor Ma-Li Wong, Department of Psychiatry, State University of New York Upstate Medical University, Syracuse, NY, USA. E-mail: WongMa@upstate.edu or Professor Julio Licinio, College of Medicine, State University of New York Upstate Medical University, Syracuse, NY, USA. E-mail: licinioJ@upstate.edu (Ph. 315-464-1682), or A/Professor Geraint Rogers, SAHMRI Microbiome Research Laboratory, Flinders University College of Medicine and Public Health, Bedford Park, SA, Australia. E-mail: Geraint.Rogers@sahmri.com.

**Conflict of interest: The authors declare no conflict of interest.**

## Supplementary methods

All procedures were approved by the South Australian Health and Medical Research Institute (SAHMRI) Ethics committee and are in accordance with the Australian Code for the Care and Use of Animals for Scientific Purposes (8th edition, 2013). All efforts were made to minimize animal suffering. Male C57BL/6J mice (wild-type, wt, n=26) aged 60 days were obtained from the SAHMR1 Bioresources Facility (Adelaide, Australia). Age-matched (*Casp1, Ifngr, Nos2*)$^{-/-}$ mice (n=30) with C57BL/6J background were generated by back-crossing mice with individual gene deletions.[1-3] After a seven-day acclimatization period, CUS mice were singly housed and control mice were group housed in transparent Plexiglas cages (Green Line IVC Sealsafe PLUS mouse, Tecniplast, Varese, Italy) in a temperature (21°C ±1°C), humidity (50%) and light (12 hour cycles, lights on at 7:00 am) controlled room, with water and food *ad libitum*. Behavioural testing was performed in the light phase of the light cycle as recommended by Castagne and colleagues.[4]

Fresh faecal pellets were collected between 10-11 am with sterile toothpicks on experimental day 41 during weighing procedures. At the endpoint of the experiment, mice were euthanized by cervical dislocation and blood was collected by cardiac puncture in EDTA coated tubes. A timeline of experimental procedures is shown in Supplementary figure 1.

*Chronic unpredictable stress*

The CUS procedure used in this study is a variation of the procedure previously described as a naturalistic model of depression in rodents.[5] The CUS protocol consisted of chronic exposure (28 days) to various randomly scheduled, low and mild intensity social and environmental stressors, applied each day during the light phase of the light cycle (except for light cycle reversal stress, which was applied during the weekend). A detailed calendar of the CUS protocol is provided in Supplementary table 2. Depending on the duration of the stressor, one

(if it lasted more than two hours) or two (if it lasted two hours or less) stressors were applied each day. The schedule was randomized weekly to maximise the degree of unpredictability and to avoid habituation, which is one of the drawbacks in modelling depression in rodents.[6] Briefly, the stressors were: a) two hours restraint in polypropylene restrainers on an open bench, b) eight hours removal of bedding and nesting material, c) eight hours of soiled bedding, obtained by adding 200 mL of autoclaved water to 100 g of bedding, d) eight hours of 45º cage tilting obtained by introducing a Plexiglas "tilter" inside the cage to allow the cage to be returned to the individually ventilated cage rack, e) two hours of predator stress, obtained by introducing in the cage a 5 ml test tube modified with ten 2 mm holes containing two fresh rat faecal pellets, f) five minutes forced swim test, performed once at the beginning and once at the end of the stress period (representing both a stressor and a behavioural test), g) sixteen hours of overnight fasting in clean cages, h) two hours of social stress, consisting in pair housing two mice from different litters in a neutral cage, i) two hours of light cycle disruption during the light phase, and j) forty-eight hours of light cycle reversal over the weekends. Sucrose preference tests, considered an index of anhedonic-like behaviour,[7] were performed weekly to assess the effectiveness of the CUS procedure.

*Behavioural testing*

Mice were submitted to open field, elevated plus maze, forced swim, and sucrose preference tests. All tests were videorecorded by a camera coupled to Ethovision XT 10 computer software (Noldus, Wageningen, Holland) for behaviour recognition and scoring.

*Open field test*

Animals were placed in the centre of a brightly lit novel arena (50cm x 50cm x 50cm) and their activity was recorded for 30 minutes by a camera mounted above the arena. Total distance (locomotor activity) and average velocity (locomotor speed) were used to quantify locomotor

activity. The number of visits (centre visits) and the total time spent in the center of the arena (centre time) were used to assess anxiety-like behaviour. The number of defecations was recorded and used as an index of emotionality,[8,9] The arena was carefully cleaned with F10 after each test session.

*Elevated plus maze*

Animals were placed in the central square (10 x 10cm) of a plus-shaped maze (1m above the ground) with two open (30 x 10cm, 1 cm-high border) and two closed arms (30 x 10cm, 20cm-high walls) for 5 minutes. The latency to enter any of the open arms and the time spent in any of the open arms were used as anxiety measures, because anxiolytic drugs decrease such parameters.[9,10] An entry in an arm required the animal to enter that arm with all four paws. The maze was carefully cleaned with F10 after each test session.

*Forced swim test*

To assess depressive-like behaviour, mice were individually placed in a clear Plexiglas cylindrical container (50cm tall, 30cm diameter) containing 30cm of water at 21°C. The amount of time spent floating, swimming and climbing (respectively <12%, 12%< x <18% and >18% activity) was automatically recorded during a 300 second test period by a camera mounted on the side of the cylinders. The percentage of activity corresponding to each behaviour was set by observing pre-recorded videos.

*Sucrose preference test*

To assess anhedonic-like behaviour, mice were individually housed and given 2 drinking bottles containing a 1% sucrose solution in drinking water for 24h to familiarize them with the novel drink. The following two days, one bottle was replaced with a standard drinking water bottle and mice were given the choice to drink from either bottle for 48 hours (training). On the fourth day (test day), the amount of liquid drunk from either bottle was recorded and the

sucrose preference was determined by calculating the percentage of the volume of sucrose drunk over the total volume of fluid drunk.[5,11]

*Adrenocorticotropic hormone* (ACTH) *and corticosterone* (CORT) *measurement*

ACTH was measured in plasma by using a competitive inhibition ELISA kit following manufacturer's instruction (Cloud-Clone Corp., Wuhan, Hubei, China). Circulating CORT was measured by using a competitive immunoassay ELISA kit following manufacturer's directions (Enzo Life Sciences, Farmingdale, New York, USA).

*Statistical analysis*

Power analysis was performed based on the effect size seen in a previous pilot study investigating the effects of simultaneous *Casp1, Nos2 and Ifngr* deficiency on total floating time in the forced swim test (our primary outcome measure). Cohen's *d* for that study was 0.84, meaning that a sample size of n=36 would result in over 80% power to detect an antidepressant-like effect at $P \leq 0.05$. Statistical analyses of the behavioural tests were performed using the Statistical Package for the Social Sciences version 23.0 for windows (SPSS, Chicago, Illinois) using a general linear model for repeated measures (repeated measures ANOVA). The effects of genotype, stress, treatment and their interaction were explored and the significance set at $P \leq 0.05$. Sphericity of the variances of the groups was assessed with Mauchly's sphericity test. Effect size was reported as partial eta-squared ($\eta^2_p$). If the stress-genotype interaction was significant, it was further assessed as described previously [12]. Statistical analyses of ELISA results were performed by two-tailed unpaired t-test.

*16S rRNA sequencing and bioinformatics analysis*

16S rRNA amplicon libraries were generated and indexed based on the Illumina Miseq 16S Metagenomic Sequencing Library Preparation protocol with modifications. Amplicons of the V4 hypervariable region of the 16S rRNA gene were generated using modified universal

bacterial primer pairs 515F (5'-TCGTCGGCAGCGTCAGATGTGTATAAGAGACAGGTGCCAGCMGCCGCGGTAA-3') and 806R (5'-GTCTCGTGGGCTCGGAGATGTGTATAAGAGACAGGGACTACHVGGGTWTCTAAT-3'), with Illumina adapter overhang sequences (underlined), as described previously (Choo *et al.*, 2015). Dual-indexed libraries were generated using the Nextera XT DNA Library Prep kit (Illumina) as a strategy for multiplex sequencing. Paired-end sequencing of the final library was performed on a 2 x 300 bp Miseq Reagent kit v3 at the David R Gunn Genomics Facility, South Australian Health and Medical Research Institute.

## Supplementary figures and table

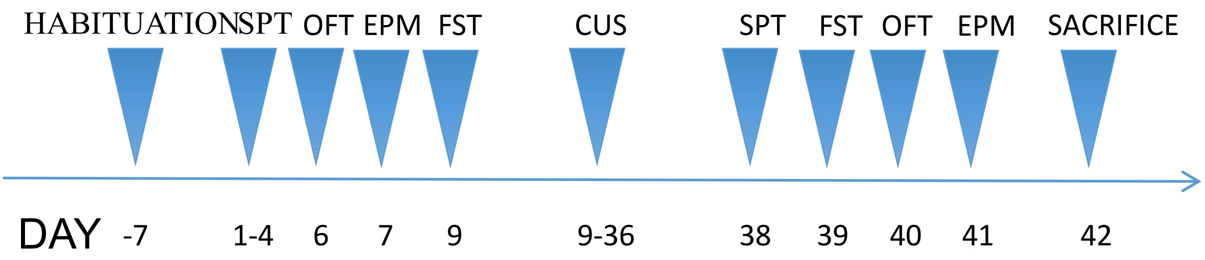


**Supplementary Figure 1.** Timeline of behavioural experiments SPT= sucrose preference test; OFT= open field test; EPM= elevated plus maze test; FST= forced swim test; CUS= chronic unpredictable stress. Faecal pellets were collected on week 0 and week 4 (following 28 days CUS).

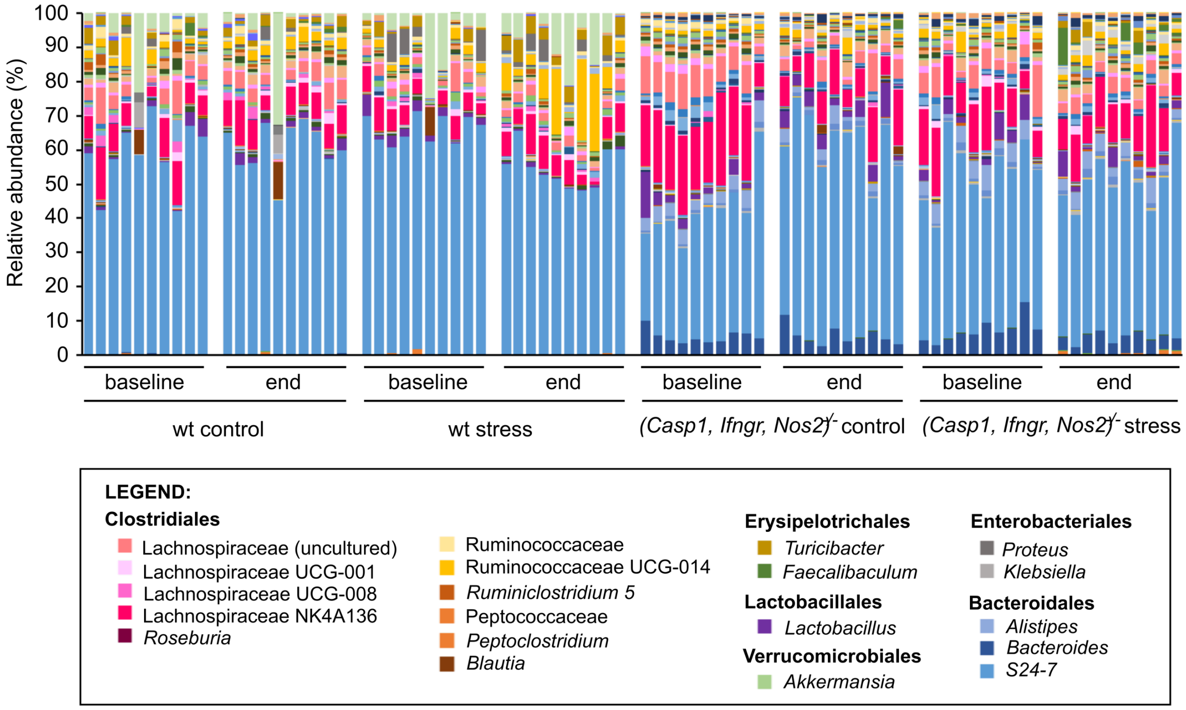


**Supplementary Figure 2.** Composition plot depicting the relative abundance of operational taxonomic unit (OTU) in faecal samples of wt and (*Casp1, Ifngr, Nos2*)$^{-/-}$ mice at baseline (week 0) and after 4 weeks of control or chronic unpredictable stress (CUS) treatment.

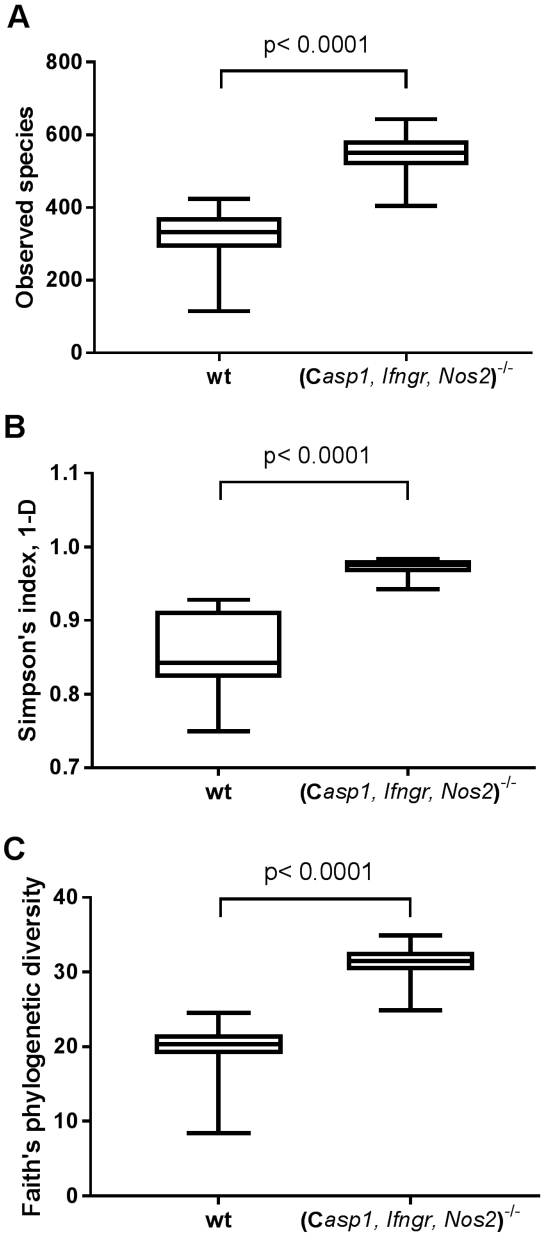


**Supplementary Figure 3.** Alpha diversity measures of microbial (A) richness (observed species), (B) evenness (Simpson's index) and (C) diversity (Faith's phylogenetic diversity) for faecal samples of wt and (*Casp1, Ifngr, Nos2*)$^{-/-}$ mice at baseline. Statistical comparison between groups were performed using the Mann-Whitney test at a significance level of 0.05.

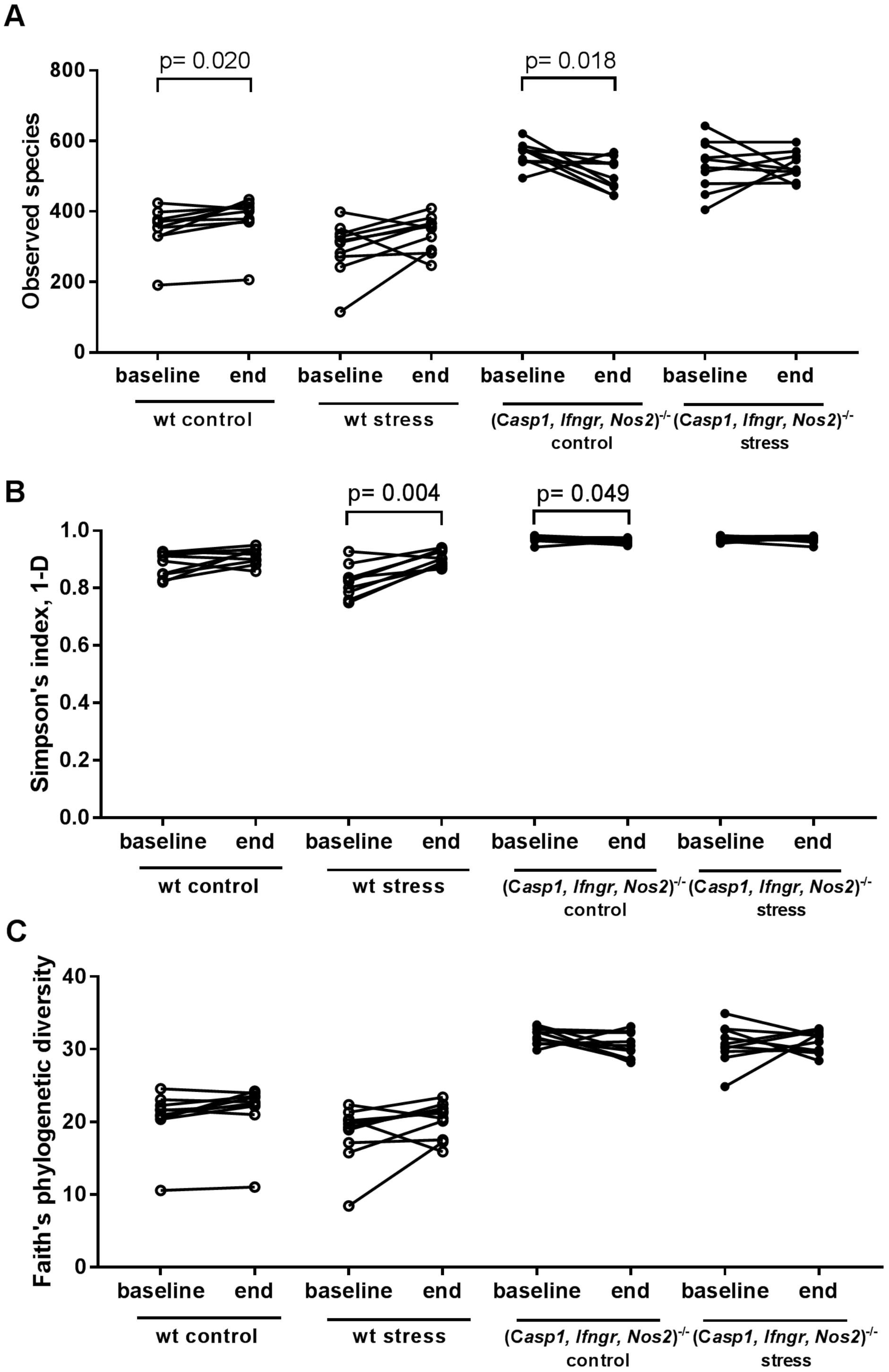


**Supplementary Figure 4.** Alpha diversity analysis of faecal samples of wt and (*Casp1, Ifngr, Nos2*)$^{-/-}$ mice at baseline and after 28 days of control or chronic unpredictable stress (CUS) treatment. Paired comparisons between timepoints for each group were performed using the Wilcoxon test at a statistical significance level of 0.05.

| TEST | MEAUSRE | wt BL (n=16) | KO BL (n=20) | wt CUS (n=16) | KO CUS (n=20) | MAUCHLY'S W | df | F-TEST | *P*-VALUE | Partial Eta Squared |
|---|---|---|---|---|---|---|---|---|---|---|
| Forced swim test | Floating (s) | 207.623 ± 6.097 | 186.102 ± 5.453 | 227.147 ± 7.895 | 187.790 ± 7.061 | 1.000 | 1,34 | G F=14.618<br>S F=4.299<br>GxS F=3.040 | G *P*=0.001 **<br>S *P*=0.046 *<br>GxS *P*=0.090 | G η2p=0.301<br>S η2p=0.112<br>GxS η2p=0.082 |
| Forced swim test | Swimming (s) | 53.533 ± 3.199 | 71.720 ± 2.862 | 45.748 ± 4.510 | 71.976 ± 4.034 | 1.000 | 1,34 | G F=25.256<br>S F=1.774<br>GxS F=2.023 | G *P*<0.001 ***<br>S *P*=0.192<br>GxS *P*=0.164 | G η2p=0.426<br>S η2p=0.050<br>GxS η2p=0.056 |
| Forced swim test | Climbing (s) | 36.618 ± 3.325 | 41.590 ± 2.974 | 25.458 ± 3.685 | 39.572 ± 3.272 | 1.000 | 1,34 | G F=5.929<br>S F=6.545<br>GxS F=3.150 | G *P*=0.020 *<br>S *P*=0.015 *<br>GxS *P*=0.085 | G η2p=0.148<br>S η2p=0.161<br>GxS η2p=0.085 |
| Sucrose preference test | Sucrose preference (%) | 85.243 ± 1.540 | 88.390 ± 1.377 | 30.457 ± 7.074 | 74.224 ± 6.327 | 1.000 | 1,34 | G F=23.331<br>S F=50.384<br>GxS F=17.485 | G *P*<0.001 ***<br>S *P*<0.001 ***<br>GxS *P*<0.001 *** | G η2p=0.960<br>S η2p=0.597<br>GxS η2p=0.340 |
| Open field test | Locomotor activity (cm) | 7744.286 ± 473.444 | 12649.357 ± 423.461 | 7756.406 ± 414.117 | 10430.527 ± 370.397 | 1.000 | 1,34 | G F=58.883<br>S F=10.852<br>GxS F=11.091 | G *P*<0.001 ***<br>S *P*<0.002 ***<br>GxS *P*=0.002 ** | G η2p=0.634<br>S η2p=0.242<br>GxS η2p=0.246 |
| Open field test | Average velocity (cm/s) | 4.306 ± 0.264 | 7.034 ± 0.236 | 4.313 ± 0.230 | 5.798 ± 0.206 | 1.000 | 1,34 | G F=58.777<br>S F=10.892<br>GxS F= 11.154 | G *P*<0.001 ***<br>S *P*<0.002 **<br>GxS *P*=0.001 ** | G η2p=0.634<br>S η2p=0.243<br>GxS η2p=0.247 |
| Open field test | Defecations (n) | 6.688 ± 0.507 | 5.700 ± 0.453 | 6.000 ± 0.782 | 9.600 ± 0.700 | 1.000 | 1,34 | G F=4.128<br>S F=7.005<br>GxS F=14.285 | G *P*= 0.050 *<br>S *P*= 0.012 **<br>GxS *P*= 0.001 ** | G η2p=0.108<br>S η2p=0.171<br>GxS η2p=0.296 |
| Open field test | Centre visits (n) | 79.500 ± 6.883 | 139.650 ± 6.157 | 70.313 ± 7.989 | 106.650 ± 7.146 | 1.000 | 1,34 | G F=35.424<br>S F=12.942<br>GxS F=4.123 | G *P*<0.001 ***<br>S *P*=0.001 **<br>GxS *P*=0.050 * | G η2p=0.946<br>S η2p=0.108<br>GxS η2p=0.108 |
| Open field test | Centre time (s) | 176.995 ± 21.473 | 187.032 ± 19.206 | 129.172 ± 17.253 | 139.128 ± 15.431 | 1.000 | 1,34 | G F=0.200<br>S F=12.583<br>GxS F=0.00 | G *P*=0.658<br>S *P*<0.001 ***<br>GxS *P*=0.998 | G η2p=0.006<br>S η2p=0.270<br>GxS η2p=0.000 |
| Open field test | Centre/total distance | 0.153 ± 0.013 | 0.179 ± 0.012 | 0.137 ± 0.013 | 0.166 ± 0.012 | 1.000 | 1,34 | G F=3.330<br>S F=2.442<br>GxS F=0.030 | G *P*=0.077<br>S *P=0.127*<br>GxS *P*=0.864 | G η2p=0.930<br>S η2p=0.067<br>GxS η2p=0.067 |
| Elevated plus maze | Open arms time (s) | 19.505 ± 4.144 | 28.492 ± 3.706 | 13.675 ± 3.045 | 23.576 ± 2.724 | 1.000 | 1,34 | G F=15.480<br>S F=10.423<br>GxS F=1.999 | G *P*<0.001 ***<br>S *P*=0.003 **<br>GxS *P*=0.166 | G η2p=0.969<br>S η2p=0.235<br>GxS η2p=0.056 |
| Elevated plus maze | Entries in any arm (n) | 21.375 ± 1.527 | 31.700 ± 1.366 | 16.250 ± 1.637 | 21.500 ± 1.464 | 1.000 | 1,34 | G F=20.348<br>S F=38.389<br>GxS F=4.210 | G *P*<0.001 ***<br>S *P*<0.001 ***<br>GxS *P*=0.048 * | G η2p=0.374<br>S η2p=0.530<br>GxS η2p=0.110 |
| Elevated plus maze | Open arms latency (s) | 20.940 ± 7.309 | 5.208 ± 6.537 | 53.140 ± 18.663 | 36.240 ± 16.692 | 1.000 | 1,34 | G F=1.464<br>S F=5.564<br>GxS F=0.002 | G *P*=0.235<br>S *P*=0.024 *<br>GxS *P*=0.966 | GxS η2p=0.217<br>GxS η2p=0.141<br>GxS η2p=0.000 |
| Elevated plus maze | Open/closed arms time ratio | 0.091 ± 0.030 | 0.233 ± 0.027 | 0.058 ± 0.015 | 0.105 ± 0.013 | 1.000 | 1,34 | G F=17.820<br>S F=13.019<br>GxS F=4.511 | G *P*<0.001 ***<br>S *P*=0.001 **<br>GxS *P*=0.041 * | G η2p=0.778<br>S η2p=0.117<br>GxS η2p=0.117 |
| Elevated plus maze | Head directed to open arms (s) | 30.255 ± 2.984 | 41.452 ± 2.669 | 21.230 ± 1.754 | 21.164 ± 1.569 | 1.000 | 1,34 | G F=5.135<br>S F=45.423<br>GxS F=6.679 | G *P*<0.030 *ssss<br>S *P*<0.001 ***<br>GxS *P*=0.014 * | G η2p=0.131<br>S η2p=0.571<br>GxS η2p=0.164 |

**Supplementary Table 1.** Statistical report of caspase 1, interferon gamma receptor and nitric oxide synthase knockout (*Casp1*, *Ifngr*, *Nos2*)$^{-/-}$ vs. wild-type (wt) mice behavioural results.

Values in columns 3-6 are means ± s.e.m.; BL=baseline; CUS=stress; df=degrees of freedom; G=genotype effect; S=stress effect; GxS=genotype x stress interaction; $\eta^2_p$=partial eta squared; *=*P*<0.05; **= *P* <0.01; ***= *P* <0.001.

| Day # | Stress Day # | Stress 1/Procedure | Stress 2 |
|---|---|---|---|
| 1 | | Sucrose preference test (habituation) | |
| 2 | | Sucrose preference test (training) | |
| 3 | | Sucrose preference test (training) | |
| 4 | | Sucrose preference test (test) | |
| 5 | | Move to individual cages | |
| 6 | | Open field test | |
| 7 | | Elevated plus maze | |
| 8 | | **START CUS** | |
| 9 | 1 | Forced swim test | 2h Restraint |
| 10 | 2 | Cage Tilting | |
| 11 | 3 | Social stress | Overnight Fast |
| 12 | 4 | Wet Bedding | |
| 13 | 5 | Predator stress | 2h Restraint |
| 14 | 6 | Light cycle reversal | |
| 15 | 7 | Light cycle reversal | |
| 16 | 8 | Sucrose preference test | |
| 17 | 9 | 2h Restraint | Predator stress |
| 18 | 10 | No Bedding | |
| 19 | 11 | 2h Restraint | Overnight fast |
| 20 | 12 | Cage Tilting | |
| 21 | 13 | Light cycle reversal | |
| 22 | 14 | Light cycle reversal | |
| 23 | 15 | Wet Bedding | |
| 24 | 16 | Sucrose preference test | Sucrose preference test |
| 25 | 17 | 2h light cycle disruption | |
| 26 | 18 | Social stress | Overnight (16h) fast |
| 27 | 19 | Cage Tilting | |
| 28 | 20 | Light cycle reversal | |
| 29 | 21 | Light cycle reversal | |
| 30 | 22 | No bedding | |
| 31 | 23 | Social stress | 2h Restraint |
| 32 | 24 | Sucrose preference test | Sucrose preference test |
| 33 | 25 | 2h light cycle disruption | Overnight Fast |
| 34 | 26 | Wet bedding | |
| 35 | 27 | Light cycle reversal | |
| 36 | 28 | Light cycle reversal | |
| 37 | | **END CUS** | |
| 38 | | Sucrose preference test | |
| 39 | | Forced swim test | |
| 40 | | Open field test | |
| 41 | | Elevated plus maze | |
| 42 | | **SACRIFICE** | |

**Supplementary Table 2.** CUS protocol calendar

**References**


1 Kuida, K. *et al.* Altered cytokine export and apoptosis in mice deficient in interleukin-1 beta converting enzyme. *Science* **267**, 2000-2003 (1995).

2 Laubach, V. E., Shesely, E. G., Smithies, O. & Sherman, P. A. Mice lacking inducible nitric oxide synthase are not resistant to lipopolysaccharide-induced death. *Proc Natl Acad Sci U S A* **92**, 10688-10692 (1995).

3 Huang, S. *et al.* Immune response in mice that lack the interferon-gamma receptor. *Science* **259**, 1742-1745 (1993).

4 Castagne, V., Moser, P. & Porsolt, R. D. in *Methods of Behavior Analysis in Neuroscience Frontiers in Neuroscience* (eds nd & J. J. Buccafusco) (2009).

5 Papp, M., Willner, P. & Muscat, R. An animal model of anhedonia: attenuation of sucrose consumption and place preference conditioning by chronic unpredictable mild stress. *Psychopharmacology (Berl)* **104**, 255-259 (1991).

6 Mineur, Y. S., Belzung, C. & Crusio, W. E. Effects of unpredictable chronic mild stress on anxiety and depression-like behavior in mice. *Behav Brain Res* **175**, 43-50, doi:10.1016/j.bbr.2006.07.029 (2006).

7 Harkin, A., Houlihan, D. D. & Kelly, J. P. Reduction in preference for saccharin by repeated unpredictable stress in mice and its prevention by imipramine. *J Psychopharmacol* **16**, 115-123 (2002).

8 Walsh, R. N. & Cummins, R. A. The Open-Field Test: a critical review. *Psychol Bull* **83**, 482-504 (1976).

9 Carola, V., D'Olimpio, F., Brunamonti, E., Mangia, F. & Renzi, P. Evaluation of the elevated plus-maze and open-field tests for the assessment of anxiety-related behaviour in inbred mice. *Behav Brain Res* **134**, 49-57 (2002).

10 Walf, A. A. & Frye, C. A. The use of the elevated plus maze as an assay of anxiety-related behavior in rodents. *Nat Protoc* **2**, 322-328, doi:10.1038/nprot.2007.44 (2007).

11 Strekalova, T. *et al.* Update in the methodology of the chronic stress paradigm: internal control matters. *Behav Brain Funct* **7**, 9, doi:10.1186/1744-9081-7-9 (2011).

12 Kinnear, P. R. & Gray, C. D. *PASW statistics 17 made simple (replaces SPSS statistics 17)*. 1st edn, (Psychology Press, 2010).
